# Monthly Summary

**Published:** 1884-10

**Authors:** 


### MONTHLY SUMMARY.

MICRO ORGANISMS.—From advance sheets of the Introductory Lecture at the opening of the fall course of the Medico-Chirurgical College, of Philadelphia, by Prof. Hugh Engel, we glean the following interesting points about micro-organisms. The address, in full, will be published in our columns, For a long time investigators were undecided whether micro-organisms, as they are comprehensively termed, belonged to the animal or the vegetable kingdon. It has now been decided that they belong to the latter. The terms *micro-organisms*, *microbes*, and *microzymes*, all mean the same thing, Micro-organisms are divided into two primary divisions: 1, HYPHOMYCETI, and 2, SCHIZOMYCETI. The first class are the pathogenic factors of external diseases, as favus, aphthæ and thrush; while the second clause the internal diseases. The schizomyceti are subdivided into the following classes:

1. COCCI or MICROCCOCCI, which are small round bodies, and embrace the cocci of phenomena, pigment cocci, and some ferment cocci.

2. BACILLI, which have a rod-like shape, whether long or short, such as the bacilli of typhoid fever and tuberculosis.

3. BACTERIA *proper* (the whole class are otten called bacteria,) such as bacterium termo, the common grade ferment.

2. VIBRIONES or VIBRIOS, those which have a wavy form, as *probably* the comma bacillus of cholera.

5. SPIRILLI, in the form of stiff screws, such as many of the micro-organisms causing decomposition.

5. SPIROCHÆ, in the shape of flexible screws and other fantastic forms, such as the micro organism of relapsing fever.

It has been calculated by Ferdinand Cohn, that if the bacterium termo were unimpeded in its propagation, and if one were to multiply into two in the first hour and these into four in the second hour, and these into eight in the third hour, and so on, the result would be 16,000,500 in the first day, and 281,000,000,000 on the second day, and that in five days' time the progeny of this little microscopical body would fill the oceans of the world, so wonderfully numerous and fertile are they. One microccoccus, one fivehundredth of a millimetre long and one one-thousandth of a millimetre thick, and of which 1,600,000,000 are required to weigh one grain, would in three days produce 50,000,000, pounds of offspring.

*Teberine* is a disease of the silk-worm that threatened to destroy the silk industry of France. Pasteuer found a bacterium in ova that were apparently healthy; all such avo were destroyed, and the threatened danger was averted.

Dr. Koch, of Berlin, furnishes some strong reasons for believing that the comma bacillus is the cause of cholera. When in Toulon, Dr. Koch made it a practice to carefully examine the discharges from, and the dead bodies of, all patients with typhoid fever, dysentery, and the like. In not one single case did he find the comma bacillus. A French sailor was in the hospital, convelescing from typhoid fever; there were no evidences of the disease remaining, he was only weak. At nine o'clock one morning he received word that he would be discharged at noon. At ten o'clock he was taken sick with cholera, and died before eleven. Within six minutes after death an autopsy was made in the presence of Koch. No morbid lesions were found, but the rice-water contents of the intestines contained innumerable commo-bacilli.

In a village in Egypt, thirty persons were taken sick in rapid succession with cholera; they were the first case in the village. It was discovered that all of these persons had been engaged the day before in washing the clothing of cholera patients. Koch found that wherever portions of clothing soiled by cholera dejections were moist, the commo-bacillus existed in abundance; but it cannot survive more than twenty-four hours without moisture.

In a village near Calcutta, several hundred persons died from cholera in the space of thirty hours, while in villages still nearer to Calcutta (where the disease was raging) no cases had occurred. It was proven that all the persons who were attacked had used water from the same tank, and it was discovered that the day before the outbreak women had been washing clothing from cholera patients in Calcutta in this tank. And, furthermore, it was discovered that wherever, in the tank, there were accumulations of decomposing wood or organic debris, there also would be found innumerable comma-baccilli. It is said that it was the recital of this remarkable experience that caused the distinguished Professor Vichow to give his sanction to the pathogenic nature of the comma-bacillus.

Dr. Koch has also discovered that the bacteria of putrefacsion are inimical to and capable of destroying the comma-bacillus, and in this connection it was noted that whenever the sewers in Toulon were cleansed or disinfected, the cholera became worse, which fact would tend to explain the reported immuity from the disease among the scavengers of Toulon and Naples.

Acids destroy the vitality of the comma-bacillus; hence we have an explanation of the fact observed during the last cholera epidemic in London, and of the recommendation made some years since by Professor Da Costa, that when the prodromic diarrhœa is treaten with sulphuric acid cholera does not develop.

It has been also observed that this bacillus cannot pass through a healthy stomach; its vitality is destroyed. It was universally noted in Egypt, Calcutta, and Toulon, that not one single case of cholera occured in an individual whose stomach had been previously healthy. In every case there had been some slight or severe derangement of the alimentary canal. In this connection, attention is called to the fact that in Europe overloading of the stomach is most common on Sundays, and the largest majority of the fresh cases of cholera would develop on Mondays.—*Med. and Sur. Reporter.*

---

TREATMENT OF SICK HEADACHE.—Dr. Richard G. Jack reports this instructive case in the *Laucet*, August 23, 1884:

Mrs. N., aged 26, married, two children, had all her adult life suffered to some extent at the periods, and also was troubled with what is commonly called "sick-headache." She was treated by various doctors in the Western States of America, and at length she sought the advise of Dr. Thomas, of New York. He came to the conclusion that the only possible relief was to be obtained by removal of the ovaries; that she was then much to broken down for the operation; but after a trip to Europe had set up her health, he would, on her return, operate. I first saw the patient in June, 1883. She was then in the second day of the period, and under one of her headaches. These begin in one side of the head, and the pain gradually increased until the patient loses consciousness, the limbs become rigid, the hands clenched, the eyes half open and turned up, and shivering fits come on every few minutes. At other times the attack takes the form of spasm of the glottis. Seeing her first in one of her severe attacks, I introduced my hypodermic needle, and injected one-sixth of a grain of morphine. I left the needle in, and in seven seconds relief was obtained; but as it was not complete, at the end of five minutes I gave the same quantity, and withdrew the needle The pain subsided, and did not return during that period, but the patient suffered from severe sickness, which lastly thirty hours. In a few days I ordered iron and quinine, with showerbaths, good food, and early hours. When the next period was expected, I gave belladonna and bromide of potassium. As soon as the period came on, I kept the patient in bed, applied a blister of the size of half-a-crown over each ovary, and ordered a morphia pessary at night. The period passed over without a sign of pain or trouble. The tonic treatment was resumed, and the patient's health was steadily improved. The next period was anticipated a day or two with the belladonna and bromide, and when the flow began she was kept in bed the first day with half a mustard leaf over each ovary. No pain; period normal. On the next occasion, feeling the restraint irksome, and forgetting the date, the patient went to a theartre on the very night of the return of the period, and at 1 A. M. I was sent for. I injected one-third of a grain of morphia; the relief was instantaneous, and by increasing the dose to half a grain the sickness dimished. I tried the addition of atropine, but without effect. By persistence in the treatment, essentially the hypodermic, the patient is freed from headache, and no longer looks sick in mind and sick in body, having regained color in her cheeks. I have repeatedly seen the same good effect in ordinary sick-headache, either from hypodermic injection of morphia or from a dose of chlorodyne.—*Med. and Sur. Reporter.*

---

PREVENTION OF IRREGULARITY.—Mr. J. R. Brownlie, L. D. S., Glas., read a paper also on the subject of "Prevention of Irregularity." He said that while a regular arrangement of the teeth as a result of nature's handiwork was common enought, Dentists were frequently called on to see the results brought about by certain other forces acting at variance with those named, giving to the teeth a crowded arrangement which was a blemish in the mouth of beauty, and which was an important factor in the production of dental caries. The age at which prevention could be carried out was a very early one, a much earlier age than Dentists usually had the opportunity of applying their knowledge. One of the earliest habits a child acquired giving rise to deformity was that of sucking the thumb. Next there was the possibility of the teeth of the first set becoming a cause of irregularity to those of the second. He also took occasion to refer to the practice of people setting no money value upon the services rendered and time occupied in consultations. An operation, he said in itself so simple that the very veriest clown could hardly blunder, was credited with a money value, and the fee readily paid, while the special knowledge which had to be acquired by years of study, and which alone enabled one to deal intelligently with the conditions of the mouth, was set at naugh too frequently as a thing not worthy of any recognition. Indefensible thoughout, the practice was one which was essentially objectionably in regard to the case of children's mouths. If a dentist knew beforehand that on his decision would rest the question of fee or no fee, it was too much to expect from human nature that his decision would be free from bias, and founded purely on the merits of the case. The temptation was too great. If it were a question of the extraction of a temporary tooth, the operator should more than likely do what was wanted done by the parent, who was quite incapable of forming any adequate opinion of its ultimate effect upon the mouth. Was it possible that under such conditions the opinion could be founded purely on the merits of the case? The position could not be defended, and there were but few of those parents or friends who interested themselves in the subject who were not capable of recognizing the risk to which the best interests of the child were exposed, and with whom a word or two of explanation was all that was needed to secure to the operator unreservedly an opportunity to act or abstain from action *secundum artem.*

---

A NEW METHOD OF REMOVING NASAL POLYPI.—Dr. William Ralph Bell says: "I take the liberty of bringing the mode of treatment before the notice of your readers which I have practiced with the very best results in several cases. It obviates any trouble from hemorrhage, which is frequently the case when the forceps or hooks is used; it is plainless and very simple. I get my patient to blow strongly through the affected nostril, closing the other with his finger. The polypos will be brought down so that it can be easily seen through the external nares; then with my hypodermic syringe charged with a solution of tannic acid in water (of the strength of twenty grains to the fluid drachm,) I pierce the polypus with the needle, and inject ten, fifteen, or twenty minums of solution, according to size of tumor. In a few days the polypus shrivels and dries up (tanned;) it comes away without any trouble or pain, and looks like a clot of dry blood, my patients usually removing it by blowing the nose or by their fingers. In only one case, that of an old lady, had I occasion to remove it myself, and in her case I think she was afraid to do so, for when I seized it with dressing forceps, I required to make no traction to bring it away.'—*Canada Med. Record.*

---

AN OVERDOSE OF CLORAL DURING PARTURITION.—To a young and healthy patient, in her third labor, one hundred grains of chloral hydrate were given, in divided doses, in the course of forty-five minutes, in mistake for three doses of fifteen grains each. The patient became heavy and drowsy, but could be temporarily roused. The pulse was between sixty and seventy per minute. The pains became slow and ineffective. Fifteen minutes after the administration of the last dose of chloral, the membranes were ruptured, and twenty-five minutes later the child was feebly expelled by natural efforts. It was excessively pale, but began to breathe and cry after moderate efforts on the part of the accoucheur, but the skin did not exhibit its natural color for several hours. The placenta soon followed the child. Two drachms of fluid extract of ergot were given, and half an ounce of brandy. An hour after the birth, the mother was in fairly good condition, and both she and the child made a good recovery.—*Boston Med. and Sur. Journal.*

---

PREVENTION OF CARIES AND IRREGULARITY.—Mr Henry Sewill, M. R. C. S., L. D. S., Eng., read a paper at a meeting of the British Dental Association on "The Prevention of Caries." He said that caries was impossible without acid, and without vitiation of the secretions of the mouth. Without due exercise in mastication teeth could not easily be kept clean, and where the function was impeded by the presence of tender teeth, these should be brought into a healthy state or extracted. Crowding of the teeth greatly prevented the constant beneficial rubbing and polishing of them, prevented the ready access of the alkaline saliva to those surfaces where acid-forming substances find their location. He was afraid they could not hope that for the sake of the teeth of posterity men would be advised to pick out big-jawed wives but they could at least seriously impress hygienists with the fact that the human jaw needed adequate use for its due development, and that no dietary could be perfect which was composed of uniformly bland and soft substances, calling for little or no mastication.

---

MIRYACHIT—INCONVENIENCE OF A DISEASE OF IMITATION. —A contemporary has the following, which might have been foreseen. The complaint is obviously a dangerous one:

"There is a new disease, called in Russia 'Miryachit,' and in Java 'Lata.' The person affected by this disease is compelled to imitate anything he sees or hears. A doctor, dining with a riend, had just explained to him the nature of the disease, when the host, pushing forward a bottle of the best 'Encore,' said: 'Try that, doctor, it's ten years old.' The doctor mixed a stiff glass, and, about half emptying it, smacked his lips, remarking, 'Tip-top, sir.' Suddenly Barney, an Irish butler, who had been present during the doctor's explanation, seized the bottle, and, filling a tumbler, emptied it at a gulp, and smacking his lips, shouted, 'Tip-top, sir.' 'What the deuce do you mean by that!' shouted the infuriated host. 'Begorra, sir,' replied Barney, humbly, 'Shure, I'm afeard I'm efflicted wud the latha.'"—*British Medical Journal.*

---

A DANGEROUS ADULTERATION OF IODOFORM.—Dr. Biel, of St. Petersburg, called attention to a commercial adulteration of iodoform with picric acid, which cannot be detected by the test of Pharmacopœia. This mixture is not only poisonous, but is explosive when rubbed in a mortar. It can be detected by the citron-yellow color it yields to a watery fiftrate. If a solution of cyanide of potassium be added to the filtrate, no reaction will follow if iodoform be pure; but if there be a trace of picric acid present, the solution will, in the course of ten minutes, become brownish red (isopurpuric acid,) and in a short time deposits an insoluble precipitate of isopurpurate of potassium.—*Deutsch Amerikan. Apotheker Zeitung.*

---

WHY NEGROES ARE BLACK.—Surgeon-Major N. Alcock has contributed to *Nature* an interesting communication as to why tropical man is black, in which he suggests that as in the lowest animals pigment-cells placed behind a transparent nerve termination exalt its vibration to the highest pitch, the reverse takes place when, as in the negro, the pigment-cells are placed in front of the nerve terminations, and that the black pigment in the skin serves to lessen the intensity of the nerve vibrations that would be caused in a naked human body by exposure to a tropical sun; that in fact, the pigment plays the same part as a piece of smoked glass held between the sun and the eye.—*Med. and Sur. Seporter.*

HOW MUCH MILK DOES AN INFANT AT THE BREAST TAKE DAILY.—Dr. J. Lewis Smith says (ARCHIVES OF PEDAITRICS, July 15) that he and Dr. Chadbourne determined the amount of milk taken by several infants, by actual weight. Taking 451.9 grains as the weight of an ounce of milk of s. g. 1.031. He concludes that a baby the first five weeks who takes the breast every two hours receives only a little more than a fluid ounce at each nursing. Of fifteen nurslings between six and ten months, the average received by each twenty-four and six-tenth fluid ounces. Hence, if the nurslings were eight in twenty-fours, three ounces were taken at each nursing; if the nursings were twelve. the quantity each time was two ounces. Under two months of age the stomach cannot receive much more than one and a half fluid ounces. At six months the infant can probably take and digest three ounces, and in the last half of the first year four ounces.

